# Functional Movement Quality of Firefighter Recruits: Longitudinal Changes from the Academy to Active-Duty Status

**DOI:** 10.3390/ijerph18073656

**Published:** 2021-04-01

**Authors:** David J. Cornell, Stacy L. Gnacinski, Kyle T. Ebersole

**Affiliations:** 1 Health Assessment Laboratory, University of Massachusetts Lowell, Lowell, MA 01854, USA; 2 UMass Movement Research Center, University of Massachusetts Lowell, Lowell, MA 01854, USA; 3 Department of Physical Therapy and Kinesiology, University of Massachusetts Lowell, Lowell, MA 01854, USA; 4 Department of Health Sciences, Drake University, Des Moines, IA 50311, USA; stacy.gnacinski@drake.edu; 5 Human Performance and Sport Physiology Laboratory, University of Wisconsin-Milwaukee, Milwaukee, WI 53211, USA; ebersole@uwm.edu; 6 Department of Rehabilitation Sciences and Technology, University of Wisconsin-Milwaukee, Milwaukee, WI 53211, USA

**Keywords:** functional movement screen, musculoskeletal injury risk, body mass index, percent body fat, tactical athletes

## Abstract

Approximately half of the injuries experienced by firefighters consist of musculoskeletal injuries (MSKIs). Functional movement quality may be associated with MSKI risk within this tactical athlete population. Previous research indicates that measures of body composition change among firefighter recruits progressing from academy training through active-duty service, but similar changes in functional movement quality have yet to be examined. The purpose of this study was to describe longitudinal changes in functional movement quality of firefighter recruits. Body mass index (BMI), body fat (BF), and Functional Movement Screen (FMS) data were collected from 26 male firefighter recruits at the onset (W1) and completion (W14) of their training academy, and at the completion of their probationary period of active-duty service (W38). After adjusting for changes in BMI and BF across time, significant changes (*p*s < 0.05) in Composite FMS scores were identified, with significant increases in from W1 to W14 and from W14 to W38, as well as an overall increase from W1 to W38. These results suggest that the development of firefighter-specific skills can decrease the MSKI risk of firefighter recruits by facilitating enhanced functional movement competencies, particularly during tasks that require single-leg movement and core strength and stability.

## 1. Introduction

According to the National Fire Protection Association (NFPA), United States (U.S.) firefighters experienced more than 60,000 injuries in 2019, with roughly half of these injuries being musculoskeletal in nature (i.e., sprains and strains) [1]. This incidence of musculoskeletal injuries (MSKIs) among these tactical athletes [2] is in agreement with the scientific literature [3], with injury rates among firefighters reported as being seven times higher than the general population [4]. Although fireground operations are inherently dangerous and physically demanding [5], and injuries occurring on the fireground are the largest contributor (39.2%), the NFPA reports that the majority of injuries (60.8%) actually occur during non-fireground activities (e.g., non-fire emergencies, physical training, etc.) [1]. Similar MSKI statistics among firefighters has also been identified, with the low-back, lower extremities, and shoulders regularly being the most commonly injured body regions [6,7,8,9,10,11,12]. Previous research indicates that these statistics are consistent among non-U.S. firefighters as well [13,14,15]. Collectively, this body of scientific literature has confirmed that the occurrence MSKIs is not considered to be confined to fireground operations [16].

Consistent with NFPA reports [1,6], previous research has also indicated that the majority of these MSKIs have occurred during patient transport and other tasks requiring lifting and pushing and pulling activities [10,13,16], with overexertion routinely being a large contributing factor [17]. Additionally, the need to improve the ergonomics and movement competency of firefighters has been emphasized in the literature [17,18,19,20]. Consequently, the NFPA has recommended that in order to safely and effectively perform the tasks associated with the occupation of firefighting, a firefighter should be able to competently perform a variety of functional movements (e.g., squatting, lunging, and overhead reaching, etc.) [21]. While not considered to be a sole predictor of injury [22], poor functional movement quality has been associated with increased risk of MSKI among tactical athletes [23], including within the firefighter population [24,25,26].

As a result, the use of a variety of screening tools, such as the Functional Movement Screen (FMS) [27,28], has increased among both researchers and practitioners attempting to mitigate MSKI risk among tactical athlete populations [29]. Previous research indicates that, elevated levels of obesity are associated with increased MSKI risk [30,31] and decreased functional movement quality within the tactical athlete population of firefighters specifically [32]. Although firefighter recruit training academies are effective in generating improvements in a variety of health and fitness measures [33,34], including changes in body composition [35], longitudinal changes in functional movement quality have yet to be examined. Therefore, before the MSKI risk associated with the functional movement quality of firefighters can be appropriately understood, and before training programs can be designed to enhance functional movement quality of firefighters, observational changes in functional movement quality, independent from changes in body composition, should be described.

Accordingly, the purpose of the current study was to describe changes in the functional movement quality of a cohort population of firefighter recruits progressing through a traditional firefighter recruit training academy and complete their probationary period as an active-duty firefighter. Preliminary results of this study have been published in abstract form only [36].

## 2. Materials and Methods

### 2.1. Participants

A convenience sample of firefighter recruits from a Midwest U.S. urban fire department were recruited via in-person informational sessions presented by study staff. Participants were considered eligible to participate if they were: (1) older than 18 years of age; (2) were not taking any prescribed medication for a symptomatic illness; (3) did not sustain an injury or have surgery on their knees, hips, or ankles in the last year; (4) were not previously diagnosed with a heart condition or experienced chest pain or dizziness during exercise; (5) had not been instructed by a physician to refrain from participating in exercise or physical activity; and/or (6) were cleared for full participation within the training academy.

Based on these criteria, all 31 male firefighter recruits, aged 24 to 43 yrs, were eligible for participation and volunteered to participate in the current study. However, 5 recruits exited the training academy before W 14, and thus, these participants were removed from the study, resulting in a total sample size of 26 firefighter recruits (30.1 ± 4.1 yrs; 179.8 ± 4.7 cm; 87.6 ± 9.7 kg). This study was approved by the Institutional Review Board at the University of Wisconsin-Milwaukee and conducted in accordance with the Declaration of Helsinki. All participants were given opportunities to ask questions regarding the study and provided written informed consent before any data were collected.

### 2.2. Study Design

This observational research study [37] characterized longitudinal changes in functional movement quality among a cohort population of firefighter recruits progressing through the same 16-week traditional firefighter academy training program and a 22-week probationary period (December to August). Specifically, data were collected at the beginning of the training academy (W1), the end of the training academy (W14), and after completing their probationary period as an active-duty firefighter (W38). Due to scheduling needs of the fire department, it was not possible to collect data during the final week of the academy training program, and thus, data collected during week 14 represented the end of the academy program.

#### Firefighter Academy Training Program

All participants completed an identical 16-week traditional firefighter academy training program within the same Midwest U.S. urban fire department. While enrolled in the academy, firefighter recruits completed training programming 8 h per day, 5 days per week. This programming is based recommendations from *The Fire Service Joint Labor Management Wellness-Fitness Initiative (WFI)*, which was jointly developed by the International Association of Fire Chiefs (IAFC) and the International Association of Fire Fighters (IAFF) [38], and is consistent with other training programs implemented in the firefighter literature [34,39,40]. Researchers of the current study did not control or influence the implementation of any training programs.

As part of the academy, 1–2 h of structured physical training to develop technical skills associated with firefighting (e.g., victim rescue and extrication, ladder raising, roof ventilation, etc.) was completed on a daily basis. In addition, structured aerobic exercise programming and total body resistance training programming, containing both major and axillary exercises, occurred each morning. General health and wellness education classes that delivered content regarding physical fitness, nutrition, and stress management, were integrated into academy curriculum as well.

After graduating from the academy, recruits then transitioned to active-duty service as a probationary firefighter and were assigned to a fire station. During this time, these probationary firefighters were required to work traditional 24-h shifts, on a rotating 3-shift schedule. Upon successful completion of this 22-week probationary period, these probationary firefighters were then considered incumbent active-duty firefighters.

### 2.3. Procedures

To limit bias, all data were collected in the same fashion and in the same conditions during each testing session. Specifically, data were collected in group testing format indoors and at room temperature in the gymnasium of the training academy facility in a designated exercise space for the training academy. Data were collected in the morning (between the hours of 1000 and 1100) with participants were wearing standard athletic clothing (i.e., athletic shorts, t-shirt, and athletic shoes). Pre-testing participant conditions (e.g., diet intake, caffeine consumption, sleep, etc.) were not controlled by researchers.

#### 2.3.1. Body Composition Data

Body mass index (BMI) and body fat (BF) measures of body composition were chosen to represent obesity-level of the firefighter recruits, which is consistent with previous literature [32,34,41]. All body composition data were collected according to standardized testing methods endorsed by the American College of Sports Medicine (ACSM) [42] and both the IAFF and IAFC [38]. In brief, height (cm) and body mass (kg) data were collected while the participant was barefoot and wearing athletic clothing using a mechanical beam scale (Health-o-Meter Professional, Pelstar LLC, McCook, IL, USA) and BMI (kg·m^−2^) was calculated for each participant utilizing these data [43]. The right triceps, pectoral, and subscapular locations were utilized to collect skinfold measures (mm) using a Lange skinfold caliper (Beta Technology, Santa Cruz, CA, USA). Using these skinfold measures, the body density of each participant was then estimated using the corresponding Jackson and Pollock three-site skinfold equation [44]. Measures of BF (%) were then estimated using the Siri equation [45]. Excellent test–retest reliability (*r* = 0.94) of this skinfold technique has been demonstrated in the literature [46] and the same experienced researcher collected all participant skinfold data across time.

#### 2.3.2. Functional Movement Quality Data

The functional movement quality of each participant was assessed using a FMS test kit (Functional Movement Systems, Chatham, VA, USA) according to previously described methods [27,28], including within the firefighter population [32,47,48]. However, before collecting an FMS data, each participant completed a 5-min dynamic warm-up, which has been previously utilized among firefighters [32]. After completing this warm-up, each FMS sub-test was given a score of 1–3 (worst-to-best): Deep Squat, Hurdle-Step, In-Line Lunge, Shoulder Mobility, Active Straight-Leg Raise, Trunk Stability Push-Up, and Rotary Stability test. These sub-test scores were summed to create a Composite FMS score. No pain was reported by participants during any FMS sub-test and no positive findings were observed during the associated shoulder and spine clearing tests [49]. Three attempts were given to perform each sub-test of the FMS and the highest score was utilized in analyses. If the sub-test was bilateral in nature, the lowest score of that respective sub-test was used for analysis. Good test–retest reliability of Composite FMS scores has been reported in the literature (pooled ICC = 0.81) [50].

### 2.4. Statistical Analyses

Multivariable mixed linear regression models, with an unstructured repeated measures covariance structure, and follow-up pairwise comparisons, were utilized to identify potential changes in the continuous Composite FMS score data (dependent variable) across time (W1, W14, W38). Due to the moderate bivariate correlation between BMI and BF (*r* = 0.55, *p* < 0.001), two separate regression models were utilized, with one model adjusting for BMI and one model adjusting for BF as time varying covariates. These models allowed for the identification of changes in Composite FMS scores while controlling for the potential confounding influence of changes in body composition on functional movement quality [32]. To examine changes in individual components of functional movement quality, repeated measures Friedman tests and follow-up Wilcoxon signed rank tests were utilized to identify potential changes in the ordinal FMS sub-test score data across time.

Normality of the continuous Composite FMS score, BMI, and BF data were confirmed via visual inspection of distribution histograms. All descriptive data are reported as mean (SD) and all statistical analyses were conducted using SAS version 9.4 software (SAS Institute, Cary, NC, USA). An alpha of 0.05 was used to determine statistical significance for all analyses.

## 3. Results

Descriptive body composition data at W1, W14, and W38 is presented in Table 1.

**Figure 1:**
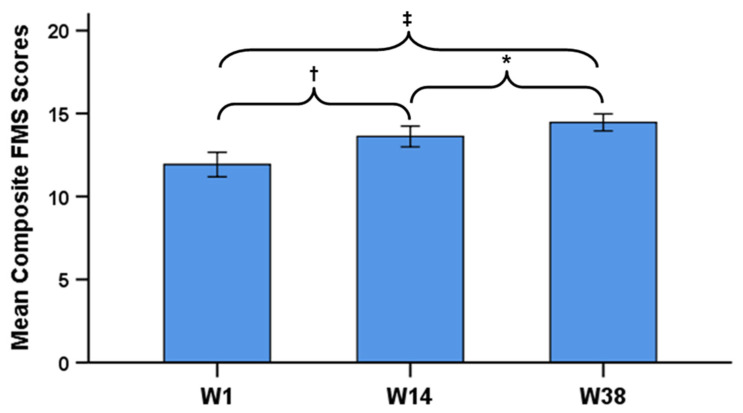
Changes in mean (± 95% confidence intervals) Composite Functional Movement Screen (FMS) scores among firefighter recruits across time. **^†^** W1 < W14; * W14 < W38; **^‡^** W1 < W38, respectively (*p* < 0.05).

After adjusting for changes in BMI across time, significant changes in Composite FMS scores were identified across time (*F*_1,25_ = 54.09, *p* < 0.001). In addition, after adjusting for changes in BF across time, significant changes in Composite FMS scores were also still identified across time (*F*_1,25_ = 43.66, *p* < 0.001). Specifically, significant increases (*p*s < 0.05) in Composite FMS scores from W1 to W14 (11.92 ± 1.83 vs. 13.62 ± 1.55, respectively), and from W14 to W38 (13.62 ± 1.55 vs. 14.46 ± 1.27, respectively), with a significant overall increase (*p* < 0.001) from W1 to W38 (+2.54), were identified in both models (Figure 1). However, only changes in BF, and not changes in BMI, had a significant influence on the changes Composite FMS scores observed across time (β = −0.12 ± 0.04, *p* = 0.005; β = −0.14 ± 0.08, *p* = 0.086, respectively).

Significant changes in Hurdle Step (χ^2^ = 22.80, *p* < 0.001), In-Line Lunge (χ^2^ = 13.77, *p* = 0.001), Trunk Stability Push-Up (χ^2^ = 7.32, *p* = 0.026), and Rotary Stability (χ^2^ = 38.08, *p* < 0.001) FMS sub-tests were identified across time. All 4 of these sub-tests significantly increased from W1 to W38 (*p*s < 0.05), with the Hurdle Step and In-Line Lunge significantly increasing from W14 to W38 as well (Table 2). However, while only the Rotary Stability sub-test significantly increased from W1 to W14, this was also the only sub-test that decreased from W14 to W38 (*p*s < 0.05). No significant changes in Deep Squat (χ^2^ = 2.60, *p* = 0.273), Shoulder Mobility (χ^2^ = 5.45, *p* = 0.066), or Active Straight-Leg Raise (χ^2^ = 4.67, *p* = 0.097) FMS sub-tests were identified across time.

## 4. Discussion

The purpose of the current study was to describe changes in the functional movement quality of a cohort population of firefighter recruits progressing through a firefighter recruit training academy and active-duty firefighter probationary periods. Based on previous research examining changes in the health and fitness of firefighter recruits, it was hypothesized that longitudinal changes in functional movement quality would follow a similar pattern previously described in the literature [33,34], which identified significant improvements in measures of health and fitness during the training academy, but subsequent significant decreases in these measures after the active-duty probationary period. Consistent with this hypothesis, results of the current study did identify significant improvements in overall functional movement quality from the beginning to end of the training academy with a 1.70 point increase in Composite FMS scores. However, in contrast to this hypothesis, a significant 0.84 point increase in Composite FMS scores was identified from W14 to W38, and from the onset of academy training to the end of a probation, and a 2.54 point increase was observed overall. These findings are particularly notable as previous research identified a minimal detectable change (MDC) metric of 2.07 for Composite FMS scores within the tactical athlete population of military service members [51]. Therefore, the total 2.54 point increase in Composite FMS scores observed in the current study would suggest that these improvements are not only statistically significant, but also are practically meaningful [52], thereby theoretically placing these individuals at a lower risk of experiencing a MSKI [23,24,25,26].

The findings of the current study are also unique as the changes in BMI and BF were statistically controlled for over time. This is an important consideration as previous research has identified significant relationships between BMI and Composite FMS scores [53,54], including among firefighter recruits [32], as well as a significant relationship between BF and Composite FMS scores [55]. Consequently, the findings of the current study indicate that the changes in functional movement quality observed across time are significant, even after controlling for the concomitant increases and decreases in BMI and BF. In addition, although both BMI and BF measures fluctuated across time, the absolute changes in BMI were minimal, and only the changes in BF significantly influenced the changes in Composite FMS scores. Therefore, these results suggest that although body composition may be inversely related to the functional movement quality of firefighter recruits, and elevated obesity-level has been positively associated with increased MSKI risk among firefighters [30,31], changes in functional movement quality are not solely driven by the changes in body composition associated with improved health and fitness during the training academy. This is noteworthy as it emphasizes the importance of implementing training programming designed to improve both functional movement quality and body composition, and in particular, BF (opposed to BMI). These findings also support the recommendations from *The Fire Service Joint Labor Management WFI*, which stress the importance of developing proper movement patterns of firefighters, in addition to enhancing general health and fitness, in an effort to mitigate MSKI risk within the firefighter population [38].

Furthermore, Composite FMS scores continued to significantly increase during the probationary period. Based on changes among FMS sub-test scores, these significant improvements in functional movement quality appear to be primarily driven by significant increases in Hurdle Step, In-Line Lunge, and Trunk Stability Push-Up sub-test scores. In addition, although a significant decrease in Rotary Stability scores was observed from W14 to W38, performance on this sub-test also significantly improved from W1 to W14. Based on upon the neuromuscular requirements to successfully perform the movement patterns associated with these 4 sub-tests [49], it is possible that firefighter recruits were capable of improving their movement competency during single-leg tasks, as well as core strength and stability, which could result in these observed improvements. While only the Rotary Stability sub-test significantly improved the during the actual academy, these results collectively imply that the combined physical (i.e., exercise and technical skill) training associated with the training academy was capable of improving functional movement quality. Coupled with the fact that the Hurdle Step, In-Line Lunge, and Trunk Stability Push-Up sub-test scores ultimately significantly increased from W1 to W38, these results imply that completing tasks associated with the occupation of firefighting may also elicit improvements in movement competency as well.

Based upon the low Hurdle Step, In-Line Lunge, and Rotary Stability sub-test scores at W1, firefighter recruits may be less proficient at performing tasks that require single-leg balance and stability and/or tasks that require core strength and stability upon entry into the training academy. As a result, it is also possible that these are the most underdeveloped movement patterns at the onset of the current study, and therefore, allowing greater opportunity for improvements in these sub-test scores. It is also possible that not only is the training that firefighter recruits receive during their academy and probationary active-duty periods are capable of improving their functional movement quality, but that proficiency at completing these occupational tasks is associated with functional movement quality. However, future research should determine if aspects of functional movement quality are associated with elements of firefighting performance and/or the ability to perform occupational tasks of firefighting in a proficient manner.

### Strengths and Limitations

The current study was the first to investigate the longitudinal changes in functional movement quality of firefighter recruits progressing from their training academy through their probationary period of active-duty service. This was also the first study to identify that changes in BF, and not BMI, significantly influenced the changes in Composite FMS scores observed among firefighter recruits across time. Thus, despite having a small sample size (*N* = 26), by statistically controlling for concomitant influences of changes in body composition, the results of the current study provide unique insight into how a firefighter recruit training academy, and the occupation of firefighting itself, may longitudinally impact functional movement quality independent of the potential confounding influence of body composition on Composite FMS scores [32,53,54,55].

However, since this was an observational study, the exercise programming utilized within the training academy was not controlled for by the researchers. Therefore, it is not possible to compare the exercise prescription parameters (e.g., intensity, duration, reps, sets, etc.) to training studies within the literature. In addition, data regarding diet, nutrition, and recreational physical activity were not collected, nor has previous research explored potential relationships between diet, nutrition, and physical activity with functional movement quality. Taken together, the lack of these data does not allow for a complete understanding of the mechanisms creating positive and negative changes in body composition and/or functional movement quality. Future research should examine the influence of different physical training programming (both exercise and technical) utilized within a firefighter recruit training academy, as well as factors related to diet, nutrition, and recreational physical activity, to better delineate potential causal mechanisms associated with longitudinal changes in body composition and functional movement quality. That said, since this was an observational study, it does provide a unique ecological perspective regarding the longitudinal changes in movement quality that typically occur within this tactical athlete population.

In addition, because all participants were members of the same firefighter academy training program, the generalizability of the results of this study are limited to training academies associated with urban fire departments in the Midwest U.S. Furthermore, since all participants were male, these results are not generalizable to female firefighters as well. This is particularly noteworthy as previous research has identified differences in scoring of the FMS between males and females [56,57,58], and therefore, it is possible that longitudinal changes in functional movement quality may differ between sexes within the tactical athlete population of firefighters. Recent research also suggests that other assessments of functional movement quality may not be influenced by body composition to the same extent as the FMS [48]. Thus, future research should examine longitudinal changes in functional movement quality, utilizing a variety of assessments of functional movement quality, among larger sample sizes of both male and female firefighters and firefighters recruited from different geographical regions and types of departments (urban vs. suburban vs. rural).

## 5. Conclusions

Results of the current study indicate that a 16-week training academy successfully improved the functional movement quality of firefighter recruits, and this functional movement quality continued to improve during the 22-week probationary period. Given relationship between poor functional movement quality and increased MSKI risk [23,24,25,26], these results suggest that the development of firefighter-specific skills during this overall period of training is capable of decreasing the MSKI risk of firefighter recruits by facilitating enhanced functional movement competencies, particularly during tasks that require single-leg movement and core strength and stability. In addition, the overall change in functional movement quality are both statistically significant and practically relevant, even after accounting for changes in body composition that occur during this firefighter recruit training period.

## Figures and Tables

**Table 1:** Body composition data across time, mean (SD).

Variable	W1	W14	W38
BMI, kg·m^−2^	27.1 (3.0)	26.7 (2.3)	27.3 (2.7)
BF, %	17.8 (4.1)	12.3 (3.2) **^†^**	14.6 (3.5)

W1, beginning of the training academy; W14, end of the training academy; W38, end of the probationary period; BMI, body mass index; BF, body fat; **^†^** W1 < W14.

**Table 2:** Changes in FMS sub-test scores across time, mean (SD).

FMS Sub-Test	W1	W14	W38
Deep Squat	1.38 (0.50)	1.50 (0.58)	1.35 (0.49)
Hurdle Step	1.38 (0.50)	1.46 (0.58)	2.0 (0.00) *, **^‡^**
In-Line Lunge	1.50 (0.58)	1.62 (0.57)	1.96 (0.45) *, **^‡^**
Shoulder Mobility	1.92 (0.80)	2.12 (0.71)	2.15 (0.68)
Active Straight-Leg Raise	2.23 (0.59)	2.19 (0.49)	2.38 (0.50)
Trunk Stability Push-Up	2.42 (0.76)	2.73 (0.45)	2.88 (0.33) **^‡^**
Rotary Stability	1.08 (0.27)	2.00 (0.00) **^†^**	1.73 (0.45) *, **^§^**

FMS, Functional Movement Screen; W1, beginning of the training academy; W14, end of the training academy; W38, end of the probationary period. **^†^** W1 < W14; * W14 < W38; **^§^** W14 > W38; **^‡^** W1 < W38, respectively (*p* < 0.05).

## Data Availability

The data presented in this study are available on request from the corresponding author.
